# Supplementary material for: Identification of Therapeutic Targets in Autism Spectrum Disorder through CHD8-Notch Pathway Interaction Analysis
Source: PLoS One. 2025 Jun 17;20(6):e0325893. doi: 10.1371/journal.pone.0325893 (PMC12173350; doi:10.1371/journal.pone.0325893)
Supplement: S1 File — This file includes: The top 20 differentially expressed genes (DEGs), including their log2 fold changes, p-values, and gene annotations. Actual gene counts or gene ratios involved in each enriched Gene Ontology (GO) term pathway. A detailed description of the processing and analysis procedures for the GSE236993 and GSE85417 datasets. (DOCX) [file pone.0325893.s001.docx]

**Top 20 DEGs with their log2 fold change, p-value, and annotation**

|  | Symbol | log2FoldChange | padj | Description |
| --- | --- | --- | --- | --- |
| 1 | A2MP1 | 9.714748 | 5.51E-19 | alpha-2-macroglobulin pseudogene 1 |
| 2 | MIR4458HG | 9.162579 | 1.29E-08 | MIR4458 host gene |
| 3 | CCL3 | 8.252203 | 6.99E-07 | C-C motif chemokine ligand 3 |
| 4 | S100A6 | 7.412158 | 2.23E-05 | S100 calcium binding protein A6 |
| 5 | COX7A1 | 7.116186 | 0.000024 | cytochrome c oxidase subunit 7A1 |
| 6 | HES2 | 6.757949 | 0.000348 | hes family bHLH transcription factor 2 |
| 7 | VCAM1 | 6.53182 | 0.00071 | vascular cell adhesion molecule 1 |
| 8 | CCL3L3 | 6.252032 | 2.15E-07 | C-C motif chemokine ligand 3 like 3 |
| 9 | GATA5 | 6.201942 | 0.00245 | GATA binding protein 5 |
| 10 | PPP1R27 | 6.02622 | 0.00294 | protein phosphatase 1 regulatory subunit 27 |
| 11 | ZNF560 | 5.5687 | 2.58E-22 | zinc finger protein 560 |
| 12 | RPS2P32 | 5.497889 | 5.56E-08 | ribosomal protein S2 pseudogene 32 |
| 13 | CILP | 5.301896 | 0.00782 | cartilage intermediate layer protein |
| 14 | HSPA2 | 5.28089 | 3.19E-36 | heat shock protein family A (Hsp70) member 2 |
| 15 | POU3F4 | 5.057644 | 1.28E-20 | POU class 3 homeobox 4 |
| 16 | NLRP7 | 5.022443 | 0.0109 | NLR family pyrin domain containing 7 |
| 17 | C5orf63 | 5.009816 | 3.53E-13 | chromosome 5 open reading frame 63 |
| 18 | NLRP2 | 4.849708 | 2.38E-66 | NLR family pyrin domain containing 2 |
| 19 | GATA2 | 4.693492 | 2.7E-08 | GATA binding protein 2 |
| 20 | CCDC152 | 4.679431 | 2.96E-08 | coiled-coil domain containing 152 |

**Actual gene counts or ratios involved in each GO termpathway**

| Ontology | ID | Description | GeneRatio | BgRatio | pvalue | p.adjust |
| --- | --- | --- | --- | --- | --- | --- |
| BP | GO:0001503 | ossification | 38/287 | 420/18800 | 8.37e-19 | 3.47e-15 |
| BP | GO:0051960 | regulation of nervous system development | 38/287 | 440/18800 | 4.08e-18 | 8.45e-15 |
| BP | GO:0050767 | regulation of neurogenesis | 33/287 | 361/18800 | 1.42e-16 | 1.96e-13 |
| BP | GO:0060284 | regulation of cell development | 38/287 | 500/18800 | 2.9e-16 | 3.01e-13 |
| BP | GO:0048568 | embryonic organ development | 35/287 | 449/18800 | 2.22e-15 | 1.84e-12 |
| CC | GO:0062023 | collagen-containing extracellular matrix | 30/289 | 429/19594 | 1.76e-12 | 6.88e-10 |
| CC | GO:0005911 | cell-cell junction | 28/289 | 497/19594 | 1.46e-09 | 2.85e-07 |
| CC | GO:0043025 | neuronal cell body | 24/289 | 482/19594 | 2.3e-07 | 2.99e-05 |
| CC | GO:0030175 | filopodium | 11/289 | 105/19594 | 4.34e-07 | 4.24e-05 |
| CC | GO:0005788 | endoplasmic reticulum lumen | 18/289 | 311/19594 | 9.35e-07 | 6.93e-05 |
| MF | GO:0019838 | growth factor binding | 17/288 | 139/18410 | 6.2e-11 | 3.39e-08 |
| MF | GO:0008083 | growth factor activity | 17/288 | 162/18410 | 7.05e-10 | 1.93e-07 |
| MF | GO:0005178 | integrin binding | 16/288 | 156/18410 | 3.16e-09 | 5.76e-07 |
| MF | GO:0050998 | nitric-oxide synthase binding | 6/288 | 13/18410 | 2.18e-08 | 2.98e-06 |
| MF | GO:0008201 | heparin binding | 13/288 | 168/18410 | 2.5e-06 | 0.0003 |

**Detailed Processing and Analysis of GSE236993 and GSE85417 Datasets**

1.GSE236993 Processing Workflow

(1)Sample Information:

This dataset includes transcriptomic data from one control group (H66) and two CHD8 allelic deletion experimental groups (CHD8A and CHD8B).

(2)Group Comparisons:

Two differential expression analyses were conducted:

CHD8A vs. H66

CHD8B vs. H66

(3)Analysis Pipeline:

Raw data were normalized using the “limma” package in R.

Differentially expressed genes (DEGs) were identified using empirical Bayes moderated t-tests.DEGs were defined as genes with an adjusted p-value (Benjamini-Hochberg False Discovery Rate, FDR) < 0.05 and absolute log₂ fold change > 1.Volcano plots and heatmaps were generated using “ggplot2” and “pheatmap” packages to visualize DEGs. Common DEGs between CHD8A and CHD8B were identified via Venn diagram analysis.

2.GSE85417 Processing Workflow

(1)Sample Information:

This dataset contains transcriptomic data from wild-type (WT) controls and CHD8 heterozygous deletion (CHD8+/−) samples.

(2)Group Comparison:

CHD8+/− vs. WT

(3)Analysis Pipeline:

Normalization and DEG identification were performed using the same pipeline as for GSE236993.Adjusted p-value (FDR) < 0.05 and |log₂ fold change| > 1 were used as thresholds for DEG selection.The expression of hub genes identified from GSE236993 was validated using this dataset.Overlapping genes between datasets were visualized with a Venn diagram and presented in heatmaps.
