# Supplementary material for: Identification of Therapeutic Targets in Autism Spectrum Disorder through CHD8-Notch Pathway Interaction Analysis
Source: PLoS One. 2025 Jun 17;20(6):e0325893. doi: 10.1371/journal.pone.0325893 (PMC12173350; doi:10.1371/journal.pone.0325893)
Supplement: Attachment — Submitted filename: Response to Reviewers.docx [file pone.0325893.s003.docx]

Manuscript ID: PONE-D-25-15242

Title: Identification of Therapeutic Targets in Autism Spectrum Disorder through CHD8-Notch Pathway Interaction Analysis

Dear Editor and Reviewers,

We sincerely thank you for your valuable feedback on our manuscript. We appreciate the time and effort you have invested in reviewing our work. In the following, we provide a detailed point-by-point response to each of the reviewers' comments. All revisions have been incorporated into the revised manuscript and are marked using track changes.

Reviewer #1

Comment 1:

Please elaborate on why the Notch signaling pathway was prioritized for interaction analysis with CHD8. Citing studies that show CHD8 indirectly or directly regulates Notch components would support this choice.

Response:

Thank you for the suggestion. In the revised manuscript, we have clarified the rationale for prioritizing the Notch signaling pathway in the context of CHD8-related ASD. Specifically, recent studies have demonstrated that CHD8, a chromatin remodeler, influences the transcription of genes involved in neurodevelopmental signaling cascades, including Notch signaling. For example, CHD8 has been reported to regulate transcriptional programs affecting neural progenitor cell proliferation and differentiation, which are also modulated by Notch activity (Weissberg and Elliott, 2021; Sood et al., 2020). Furthermore, Sugathan et al. (2014) identified Notch pathway genes as CHD8 targets in human neural progenitors using ChIP-seq data. This evidence supports our hypothesis that CHD8 mutations may disrupt neurodevelopment by modulating Notch pathway genes, prompting our focus on CHD8–Notch interactions.

Accordingly, we have updated the Introduction (Page 3, Paragraph 2) with this rationale and cited the relevant studies to justify our pathway selection.

Comment 2:

Clarify whether adjusted p-values (FDR) were used to define DEGs. If not, please re-analyze using adjusted values to reduce false positives. Include a summary table of the top 20 DEGs with their log2 fold change, p-value, and annotation.

Response:

We thank the reviewer for the valuable comment. As requested, we confirm that adjusted p-values using the Benjamini-Hochberg False Discovery Rate (FDR) correction were applied in our analysis to define differentially expressed genes (DEGs), in order to minimize the rate of false positives. The threshold for DEG selection was set at adjusted p-value (FDR) < 0.05 and |log₂ fold change| > 1.

To address the reviewer’s suggestion, we have provided a supplementary summary table listing the top 20 DEGs, including their gene symbols, log₂ fold changes, adjusted p-values (FDR), and functional annotations. This table has been added as Supplementary Table S1 in the revised manuscript and submitted as an Excel file alongside the revision.

Comment 3:

Add labels for representative genes on the volcano plots (e.g., IGF2, FN1, CXCR4). Include color scales and clustering dendrograms in the heatmaps for better interpretability.

Response:

We appreciate the reviewer’s helpful suggestion. In response, we have revised the volcano plots (Fig. 1A and Fig. 4A) to include labels for representative genes, such as IGF2, FN1, and CXCR4, to improve interpretability. In addition, we have enhanced the heatmaps (Fig. 1D and Fig. 4C) by adding hierarchical clustering dendrograms, which provide clearer insights into gene expression patterns and group relationships. These updated images have been re-uploaded.

Comment 4:

In Figure 2, please provide actual gene counts or ratios involved in each GO term/pathway.

Response:

We thank the reviewer for the constructive suggestion. In response, we have provided the actual gene counts and corresponding gene ratios for each enriched GO term and KEGG pathway shown in Figure 2. These data have been compiled and included as Supplementary Table S2 in the revised submission.

Comment 5:

While several small-molecule compounds (e.g., AMD3100 and IGF-1R inhibitors) are identified, the therapeutic implications for ASD are not well explained.

Response:

We thank the reviewer for the insightful comment regarding the therapeutic implications of the identified small-molecule compounds. In the revised Discussion section, we have expanded our explanation to better clarify the potential mechanisms by which compounds such as AMD3100 and IGF-1R inhibitors may exert therapeutic effects in ASD. We also referenced supporting studies to contextualize their roles in modulating pathways related to neurodevelopment and ASD-related phenotypes.

Comment 6:

The manuscript is generally understandable, but contains grammatical and stylistic issues. Consider professional English language editing to improve clarity and scientific tone.

Response:

We have carefully revised the entire manuscript for grammar, clarity, and scientific tone.

Reviewer #2

Comment 1:

The specific processing and analysis process of the datasets GSE236993 and GSE85417 mentioned in the article is somewhat brief. It is suggested to provide a detailed description of each step of data processing in the supplementary materials.

Response:

Thank you for the suggestion. we have added a detailed description of the specific data processing and differential expression analysis steps for both datasets (GSE236993 and GSE85417) in Supplementary Material 3. This includes sample grouping, statistical thresholds, and DEG selection criteria, thereby improving the transparency and reproducibility of our methodology.

Comment 2:

The methods section is not detailed enough, and the statistical analysis section lacks.

Response:

we have revised the Methods section to include a more comprehensive description of the statistical approaches used throughout the study, including the software version, statistical tests, normality assumptions, and significance thresholds

Comment 3:

All the abbreviations should be explained when used the first time in the manuscript. In addition, if you can avoid any of the abbreviations, it is preferred to write only full text.

Response:

We have reviewed the manuscript and ensured that all abbreviations are defined upon first use. Where appropriate, we have replaced abbreviations with full terms for clarity.

Comment 4:

It will be more relevant if the authors can verify their key observations in vivo and in vitro with their own samples.

Response:

We acknowledge this limitation and have added a paragraph in the Discussion section addressing the lack of experimental validation. We have stated our plans to conduct in vitro validation of IGF2 and CXCR4 expression levels in patient-derived samples in future work.

Comment 5:

The discussion section is relatively brief, and it is suggested to have a more detailed discussion on the functions of each key gene, especially how they play a role in the Notch signaling pathway and neural development.

Response:

We thank the reviewer for the insightful suggestion. In response, we have expanded the Discussion section to provide a more detailed analysis of the functional roles of each key gene identified (IGF2, CXCR4, FN1, COL11A1, ITGA6, LOX, and FBN2), with a particular focus on their involvement in the Notch signaling pathway and neurodevelopment.

Comment 6:

The authors should elaborate the difference between the current manuscript and previous similar published papers, and explain the advantages of current study.

Response:

We appreciate the reviewer’s comment. In response, we have added a paragraph to the Discussion section clarifying how our study differs from previous research and emphasizing its unique contributions. Specifically, we highlight our integrative analysis of CHD8-related transcriptomic datasets, focused dissection of Notch signaling pathway interactions, and the identification of convergent hub genes with therapeutic relevance. These aspects distinguish our study from earlier works and provide novel insights into the molecular mechanisms of ASD.

Comment 7:

Multiple grammar mistakes. English needs to be improved.

Response:

As noted above, the manuscript has undergone full proofreading by a native English-speaking editor. We believe the revised version addresses all linguistic concerns.

Please let us know if any additional revisions are required. Thank you again for your helpful feedback.

Sincerely,

Chao Bao, on behalf of all authors

Corresponding author

Email: drbaochao@163.com

ORCID: https://orcid.org/0000-0002-0982-8168
